# Bridging local and scientific knowledge for area-based conservation of useful plants in Colombia

**DOI:** 10.1007/s13280-023-01921-5

**Published:** 2023-10-12

**Authors:** Laura Kor, Mateo Fernández-Lucero, Diego Arturo Granados Flórez, Terence P. Dawson, Mauricio Diazgranados

**Affiliations:** 1 Research Department, Royal Botanic Gardens, Kew, Richmond, TW9 3AE UK; 2 Department of Geography, King’s College London, Bush House NE, London, WC2B 4BG UK; 3 Instituto de Investigación de Recursos Biológicos Alexander von Humboldt, Villa de Leyva, Colombia; 4 Grupo de Investigación MOPUB, Facultad de Artes, Universidad de Ciencia y Desarrollo UNICIENCIA, Bogotá, Colombia; 5 International Plant Science Center, The New York Botanical Garden, 2900 Southern Boulevard, Bronx, NY 10458 USA

**Keywords:** Conservation prioritisation, Ethnobotany, Important Plant Areas, Socio-ecological systems, Sustainable use, Useful plant species, Prioridades de conservación, Etnobotánica, Áreas Importantes para Plantas, Sistemas socio-ecológicos, Uso sostenible, Especies de plantas útiles

## Abstract

While the importance of interdisciplinary approaches is increasingly recognised in conservation, bridging knowledge systems across scales remains a fundamental challenge. Focusing on the Important Plant Areas (IPA) approach, we evaluate how complementing scientific and local knowledge can better inform the conservation of useful plants in Colombia. We worked in three municipalities to investigate knowledge on useful plant richness, species composition and use types, as well as perceptions on area-based plant conservation approaches. Participatory focus groups and ethnobotanical walks-in-the-woods were undertaken with local communities, while scientific data were represented by occurrence records from global data aggregators and digitised collections. A total of 1190 species with human uses were reported. Combining knowledge systems provided the richest understanding of useful plants but the relative contribution of each system varied between study areas, influenced by the history of scientific studies, socio-ecological context and study design. Meanwhile, local perceptions of how conservation areas should be selected differed from global IPA criteria. These results show that working with local communities can improve biological understanding for spatial conservation planning. Additionally, participatory approaches must move beyond community-based conservation and data collection, to inform the design of global conservation programmes.

**Supplementary Information:**

The online version contains supplementary material available at 10.1007/s13280-023-01921-5.

## Resumen

Si bien la importancia de los enfoques interdisciplinarios se reconoce cada vez más en la conservación, articular los sistemas de conocimiento a través de sus escalas sigue siendo un desafío fundamental. Centrándonos en el enfoque de Áreas Importantes para Plantas (AIP), evaluamos cómo la integración entre conocimiento científico y local puede mejorar la información para la conservación de las plantas útiles en Colombia. Trabajamos en tres municipios para investigar el conocimiento sobre la riqueza de plantas útiles, la composición de especies y los tipos de uso, así como las percepciones sobre los enfoques de conservación de plantas basados en áreas. Se llevaron a cabo grupos de enfoque participativos y caminatas etnobotánicas en el bosque con las comunidades, mientras que los datos científicos se representaron mediante registros de ocurrencia de agregadores de datos globales y colecciones digitalizadas. En total se reportaron 1.190 especies con usos humanos. La combinación de sistemas de conocimiento proporcionó la comprensión más rica de las plantas útiles, pero la contribución relativa de cada sistema varió entre las áreas de estudio, influenciada por la historia de los estudios científicos, el contexto socioecológico y el diseño del estudio. Por otra parte, las percepciones locales sobre cómo se deben seleccionar las áreas de conservación difirieron de los criterios globales de las AIP. Estos resultados muestran que trabajar con las comunidades locales puede mejorar la comprensión biológica para la planificación de la conservación espacial. Además, los enfoques participativos deben ir más allá de la recopilación de datos y la conservación basada en la comunidad, para instruir el diseño de programas de conservación global.

## Introduction

It is increasingly acknowledged that for conservation efforts to succeed, social–ecological systems (SES) approaches are required which view human and natural systems as interdependent and co-evolving (Díaz et al. 2015; Reyes-García and Benyei 2019). By promoting the integration of indigenous and local knowledge with scientific methods, this interdisciplinary lens enables the recognition of human impacts on landscapes and draws on the potential of diverse knowledge systems to solve environmental challenges (Charles 2021). However, global biodiversity conservation continues to be largely based on Western scientific knowledge, dominated by area-based approaches. This has been reinforced by the Post-2020 Global Biodiversity Framework (GBF) which aims for at least 30% of land and sea to be under conservation management by 2030 (CBD 2022).

Area-based conservation is often based on priority mapping, with biogeographical methods used to identify where and how to efficiently achieve conservation goals. As this relies on accurately understanding the distribution of target species, data gaps such as in species occurrence records and conservation status have long been highlighted as a limiting factor in evidence-based conservation (Holland et al. 2012). Priority mapping also faces epistemological criticisms. Wyborn and Evans (2021) argue that at the global scale, it removes local context and creates barriers to incorporating different knowledge systems. This mostly stems from the need for specific quantitative data which can hinder interdisciplinary collaboration, create regional and taxonomic bias, and perpetuate the dominance of scientific methods. While priority mapping has made many contributions to conservation, the need to integrate multiple knowledge systems and spatial scales is clear (Chaplin-Kramer et al. 2022).

With the definition of knowledge often contested, we use the Intergovernmental Platform on Biodiversity and Ecosystem Services (IPBES) definition of scientific knowledge as “typically generated in universities, research institutions and private firms …[and] typically associated with the scientific method”. Meanwhile indigenous and local knowledge (ILK) is defined as “know-how accumulated across generations, which guide human societies in their innumerable interactions with their surrounding environment”, with local knowledge (LK) considered a subset of this (Díaz et al. 2015). The IPBES Conceptual Framework provides a transdisciplinary structure to link nature and people. This builds on several decades of developing SES approaches, including participatory approaches for community-based conservation and natural resource management (Milner-Gulland and Mace 1998). Emerging evidence indicates that achieving both positive biodiversity and livelihood outcomes requires bridging multiple sources of knowledge and conservation practice, while understanding the range of relevant motivations involved (Charles 2021). However, the IPBES framework authors acknowledge the continued tendency to use scientific knowledge to validate ILK, with Tengö et al. (2014) highlighting the need to recognise complementarity. In addition, research gaps remain in how community conservation can be linked to larger-scale initiatives such as designing global programmes and informing conservation planning.

Ethnobotany—the study of local people’s categorisation, understanding and use of plants—has a long history as an interdisciplinary field, involving methods ranging from anthropology to economics (Martin 1995). It naturally fits within an SES lens, with the role of ethnobotany, and ethnobiology more generally, growing in conservation as we increasingly acknowledge the reliance of billions of people on wild species and the importance of incorporating plural knowledge sources for their sustainable use (IPBES 2022). While this is a positive shift, it has highlighted an important challenge facing ethnobiology. Namely, how to remain place-based and rooted in ILK, while matching the larger scales which are relevant to enrich and engage with national and global conservation policies and planning (Fernández-Llamazares 2023).

As such, large-scale scientific methods have been increasingly applied to useful plant conservation. This includes biogeographic analyses, global checklists and datasets (Khoury et al. 2019; Diazgranados et al. 2020; Pironon et al. 2020; Kor et al. 2022). Notably, socially, economically or culturally valuable plant species were included as potential triggers of Important Plant Areas (IPA) in 2017, a global programme to identify the best sites for plant conservation (Darbyshire et al. 2017). These data-driven approaches have the potential to suffer from aforementioned limitations such as biases in occurrence records, gaps in extinction risk assessments and difficulties incorporating plural knowledge systems. However, while based on globally consistent criteria and scientific approaches, IPAs are distinct from general definitions of protected areas due to their inclusion of useful species, potential application at different scales and governance levels and in not automatically conferring legal protection (Darbyshire et al. 2017). This recognises the human element of conservation and allows for flexibility in methodological and management approaches, thereby providing a potentially useful framework to investigate the integration of local and scientific knowledge in area-based conservation involving useful plants.

Colombia is one of the most bioculturally diverse countries on earth (Loh and Harmon 2005). Nature is deeply connected to the country’s traditions (Carrizosa-Umaña 2014) and it supports the second highest diversity of plants in the world, including over 7000 species with reported uses (Arbeláez-Cortés 2013; Diazgranados et al. 2022a). Following the 2016 Peace Agreement in Colombia, national post-conflict plans were strongly linked to conservation and bioeconomic development (DNP 2018). However, deforestation rates have since increased and a high proportion of initiatives involving wild plant use are unsustainable (Kor et al. 2021; World Resources Institute 2021).

Despite the launch of the Tropical IPA (TIPAs) programme in 2015 and publication of a specific Colombian methodology, confirmed IPAs are yet to be identified in the country (Diazgranados and Castellanos-Castro 2018). Identification of Colombian IPAs for useful plants is now ongoing and would allow application of a global conservation programme while acknowledging the livelihood and cultural importance of biodiversity (Kor et al. 2022; Kor and Diazgranados 2023; Plantlife 2022). To date, most countries have used traditional scientific data and methods in IPA identification (Blasi et al. 2011; Hamidah et al. 2020), with limited examples of selection based on useful plants. As with priority mapping in general, data gaps in species occurrences have proved a key limitation and this is acknowledged as a challenge for Colombia (Holland et al. 2012; Diazgranados and Castellanos-Castro 2018).

Here, we focus on Colombia and the area-based IPA approach to evaluate how combining local and scientific knowledge and priorities can enrich understanding and conservation of useful plants, i.e. species for which uses by humans can be reported. We take a case study approach focusing on the three pilot area municipalities of the Useful Plants and Fungi of Colombia Project (UPFC), an interdisciplinary project which aimed to develop pathways to enhance nature’s contribution to people (Diazgranados et al. 2022b). We investigate how data collected with local people and from scientific sources compare in terms of the following: (1) richness and composition of useful plants, (2) knowledge on plant use types and (3) approaches to area-based plant conservation.

## MATERIALS AND METHODS

### Methodological approach

**Fig. 1:**
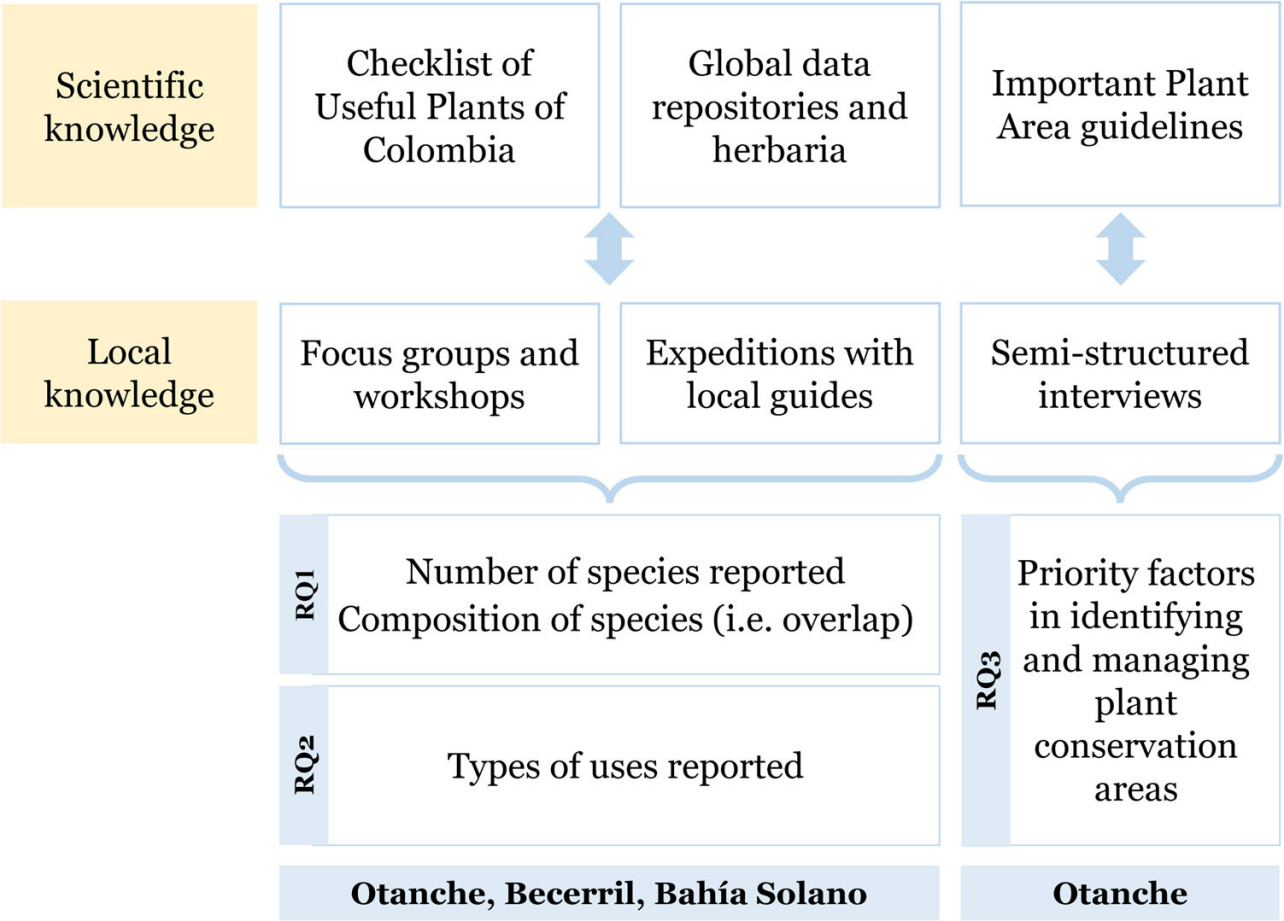
Methodological approach used to gather data on useful plants based on global- and national-level scientific sources and local knowledge

We used a mixed-methods approach to collect data across and within the scientific and local knowledge systems (Fig. 1). Our research questions draw on commonly applied criteria of IPAs which require occurrence data to identify the sites that support the following: (A) threatened species and (B) exceptional diversity (Darbyshire et al. 2017). An understanding of plant uses is relevant as, among other factors, these criteria can be triggered by the presence of individual, threatened or range-restricted plant species of socio-economic importance (A), and for holding an exceptional number of socially, economically or culturally valuable species (B).

### Study area

**Fig. 2:**
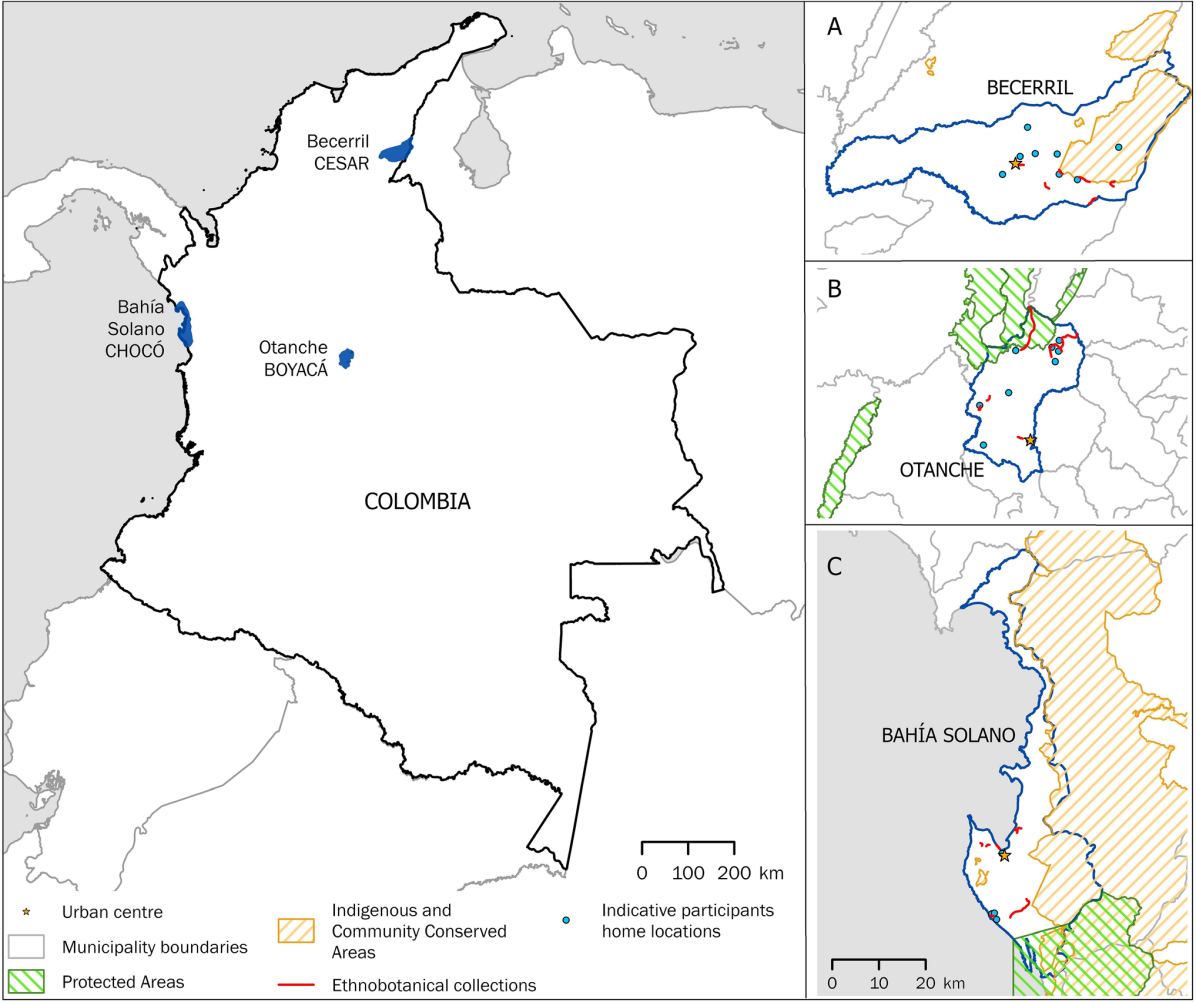
Map of pilot areas and the locations of field data collection

As our case study areas, we focused on the municipalities of Otanche (Boyacá department), Bahía Solano (Chocó department) and Becerril (Cesar department) (Fig. 2). These were chosen as the pilot areas of the UPFC following a systematic selection process with environmental, social, governance, business and economic criteria. While each municipality supports different ecosystems and cultures, all are biodiverse areas and have recently been affected by armed conflict, a factor shown to affect the sustainability of biological resource use (Diazgranados et al. 2022b; IPBES 2022).

Located in the eastern range of the Colombian Andes, Otanche has an extent of 512 km^2^ and topography ranging from 200 to 1600 m above sea level (m a.s.l.). The dominant natural ecosystem is sub-Andean forest and includes the southern extent of the Serranía de las Quinchas Natural Park and Key Biodiversity Area (KBA). Over 1000 plant species have been reported in the area and studies on useful plants have been recently published (Bohórquez Osorio et al. 2020). The primary economic activities are agriculture and mining, with 98.7% of the population in informal employment. Approximately half of Otanche’s 6997 inhabitants live in a growing urban centre (Otanche town), with the remaining 3640 spread across 43 rural districts (or *veredas*). Nearly all of Otanche’s population (99.9%) does not identify with a minority ethnic group (DANE 2019).

Bahía Solano is on the Pacific Coast of Colombia, covering 1667 km^2^. It is within the Chocó Biodiversity Hotspot, one of the most biodiverse areas globally, dominated by neotropical rainforest and coastal mangroves. A high proportion of the population belong to ethnic minorities, with 86.6% identifying as black or Afro-Colombian and 9.1% as indigenous (DANE 2019). GDP per capita in the municipality is well below the national average, with livelihoods primarily based on small-scale agriculture and fishing, alongside a small but growing ecotourism industry (DANE 2019).

Much of the 1144 km^2^ area of Becerril has been converted from tropical dry forest (TDF) to livestock grazing, oil palm plantations and open-pit mining, reflecting the main economic activities. TDF is a relatively understudied ecosystem, with ethnobotanical studies of the area only very recently undertaken (Ávila et al. 2022). A significant proportion of the population in Becerril identify as Afro-Colombian (14.7%) and indigenous (10.0%), with three indigenous reserves of the Yukpa people in the municipality (DANE 2019).

### Municipality-level local knowledge

An interdisciplinary research team undertook socio-ecological data collection between April 2021 and July 2022. In the planning phase of the UPFC, research permissions were obtained, and fieldwork was planned in collaboration with local development organisations and community leaders. This involved Boyapaz in Otanche (a regional development and peace programme), APSACESAR and Envol Vert in Becerril (organisations promoting sustainable development in agricultural areas) and self-organised *consejos comunitarios* as well as two local organisations in Bahía Solano (the John von Neumann Institute of Environmental Investigation of the Pacific and the Botanic Garden of the Pacific). The research design was in accordance with the ethical procedures of the Royal Botanical Gardens, Kew, and King’s College London. We obtained written Free, Prior and Informed Consent (FPIC) from each participant (Supplementary Information).

Our field data collection involved participatory focus groups and ethnobotanical expeditions in all three municipalities. Due to travel restrictions imposed by Covid-19 and national security concerns during the period of our research, further semi-structured interviews were conducted only in Otanche (Fig. 1). Participants were recruited through targeted sampling. This was appropriate to ensure that participants would contribute relevant knowledge on useful plants and as probability sampling is not possible for focus groups (Newing et al. 2011). Suitable participants were identified with the support of our local partner organisations with subsequent snowball sampling.

Initial focus groups conducted in 2021 involved participants free listing plants with current or traditional uses (Martin 1995; Fernandez Lucero et al. 2022). Participants with strong knowledge and interest were further recruited as local guides in ethnobotanical expeditions. In contrast to focus groups, these “walks-in-the-woods” represent an in situ ethnobotanical data collection method, shown to have higher data reliability but allowing for more restricted participation (Thomas et al. 2007). These walks were conducted across three expeditions from April to December 2021 to document and collect plants indicated as useful by the guides. A database of all plant names and uses mentioned in focus groups and expeditions was formed, followed by cleaning, standardisation and taxonomic identification to determine the accepted species names. Carried out by Fernandez Lucero et al. (2022), this included triangulation between focus group and collection results, published and herbaria sources, and consultation with local guides and ethnobotanical experts.

### Global- and national-level scientific knowledge

The Checklist of Useful Plants of Colombia (CUPC) was published as part of the UPFC Project. Compiled from a range of sources including datasets, scientific articles, books and technical reports, 7472 plant species in Colombia are listed with reported uses (Diazgranados et al. 2022a). We used this to define which plant species are considered useful based on scientific knowledge.

To determine which species occur in the study areas, we downloaded georeferenced occurrence records from the Global Biodiversity Information Facility (GBIF 2022), Botanical Information and Ecology Network (BIEN 2022), and the virtual herbarium of the Jardín Botánico de Bogotá (JBB Herbarium 2022). Data were taxonomically reconciled against the World Checklist of Vascular Plants v.5.0 (WCVP 2021), combined, cleaned including removal of duplicates and clipped to the extent of each municipality.

### Important Plant Area approach

Interviews were conducted in Otanche in July 2022. As part of a broader semi-structured interview on useful plants, this introduced the concept of IPAs to gain an understanding of participants’ perception of the programme and priorities for plant conservation and management. The interview guide was designed to be flexible and was piloted in an earlier fieldtrip with adjustments made (see Supplementary information). Field notes were taken during focus groups and interviews.

### Data analysis

#### Richness and composition

The number of species reported from scientific and local knowledge sources was totalled in each study area to compare the differences in species richness. Venn diagrams were plotted and a similarity index was calculated to indicate the level of resemblance. The Szymkiewicz–Simpson similarity index (Si), or overlap coefficient, is a metric to compare species presence–absence between two samples. Unlike other more commonly used matrices, it allows for samples where species richness largely differs by giving more weight to the smaller sample size (*b* or *c* in the equation) (Simpson 1960):

$${Si}=\frac{a}{a+{\mathrm{min}}(b,c)}$$

where *a* the number of species shared by both samples; *b* and *c* number of species occurring only in the first and only in the second sample, respectively.

**Fig. 3:**
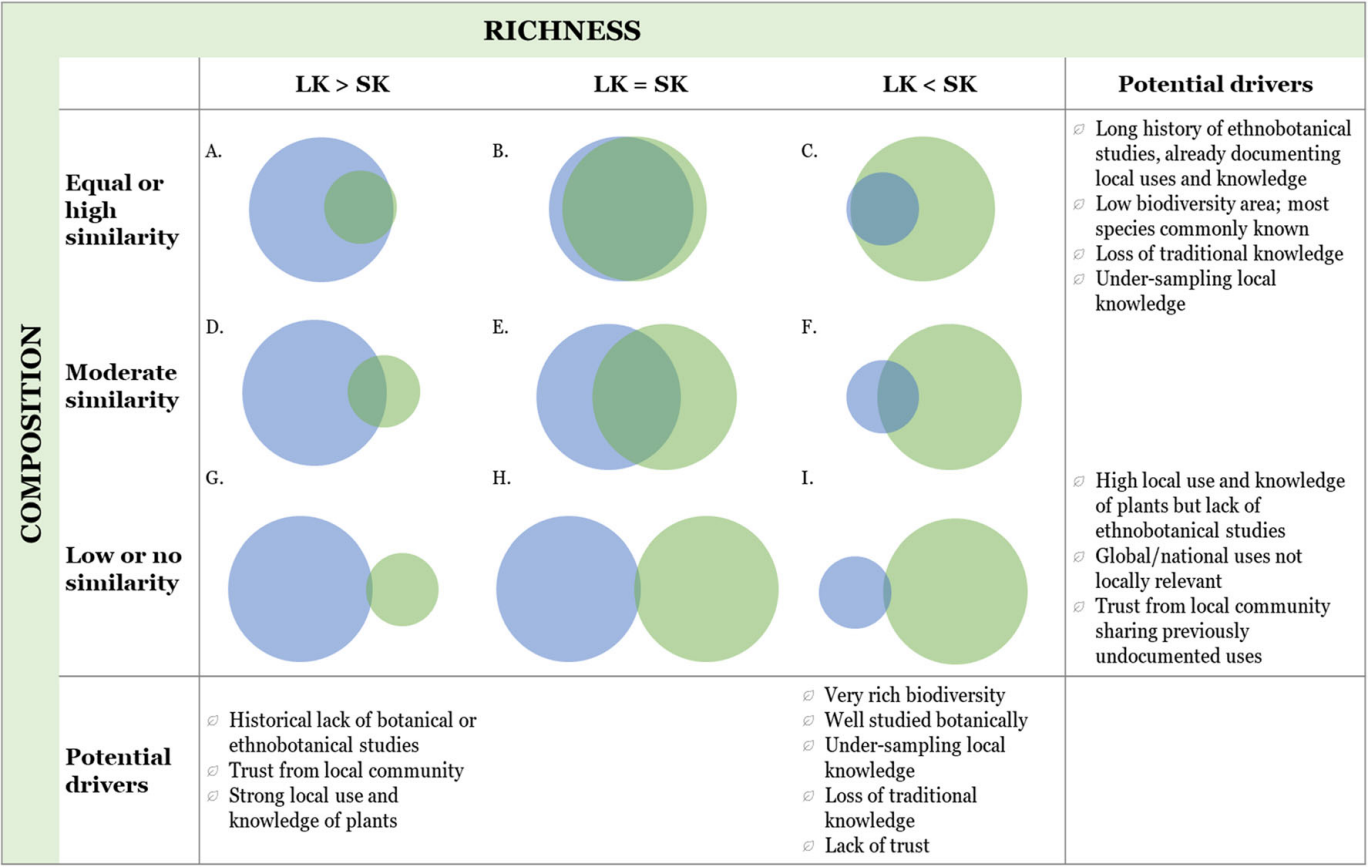
Interpretative key of potential drivers for similarities and differences on richness and composition between local ecological knowledge (LK, blue) and global- and national-level scientific knowledge (SK, green) data sources. Size of circles are proportional to number of species and overlap indicates proportion shared between the two data sources

We developed a key to support interpretation of results for each municipality. All potential combinations of relative richness and composition between the knowledge sources were identified, alongside potential reasons (Fig. 3). While results are context-specific, the table helped to direct the route of investigation into municipality-specific drivers. As this is to our understanding the first known comparative study of useful plant data between scientific and local knowledge sources, it is hoped that this key can act as reference for future studies to help identify the research gaps.

#### Use types

Plant uses were grouped within ten categories applied in the World Checklist of Useful Plant Species (Diazgranados et al. 2020), based on the Economic Botany Data Collection Standard (EBDCS) (Cook 1995) (Supporting Information). We calculated the proportion of plant species within each of these categories from scientific and local knowledge sources and within each municipality. Qualitative thematic analysis of existing literature and socio-ecological context was undertaken by the authors to contextualise each area.

#### Important Plant Area approaches

Interview data from Otanche were assessed to draw out key themes relating to the following: (1) prevalence of plant use, (2) factors which should be prioritised in IPA identification and (3) how they should be managed. Descriptive statistics were used to assess answers to closed questions while answers to open questions were coded and qualitatively assessed.

Data management, analyses and visualisation were conducted in R v.4.2.0 (R Core Team 2022) using the *tidyverse* meta-package (Wickham et al. 2019) and the packages *CoordinateCleaner* (Zizka et al. 2019), *VennDiagram* (Chen 2022) and *ggplot2* (Villanueva and Chen 2019). Mapping was conducted in ArcGIS Pro 2.8.0 (Esri Inc., 2021).

## Results

Seven focus groups were held across the municipalities of Otanche, Becerril and Bahía Solano, with a total of 95 participants (53, 23, and 19, respectively; 37 male, 58 female). These were complemented by ethnobotanical expeditions involving 51 local guides, conducted over a total of two weeks per municipality. Combined results from these local knowledge sources reported 545 unique useful plant species.

**Fig. 4:**
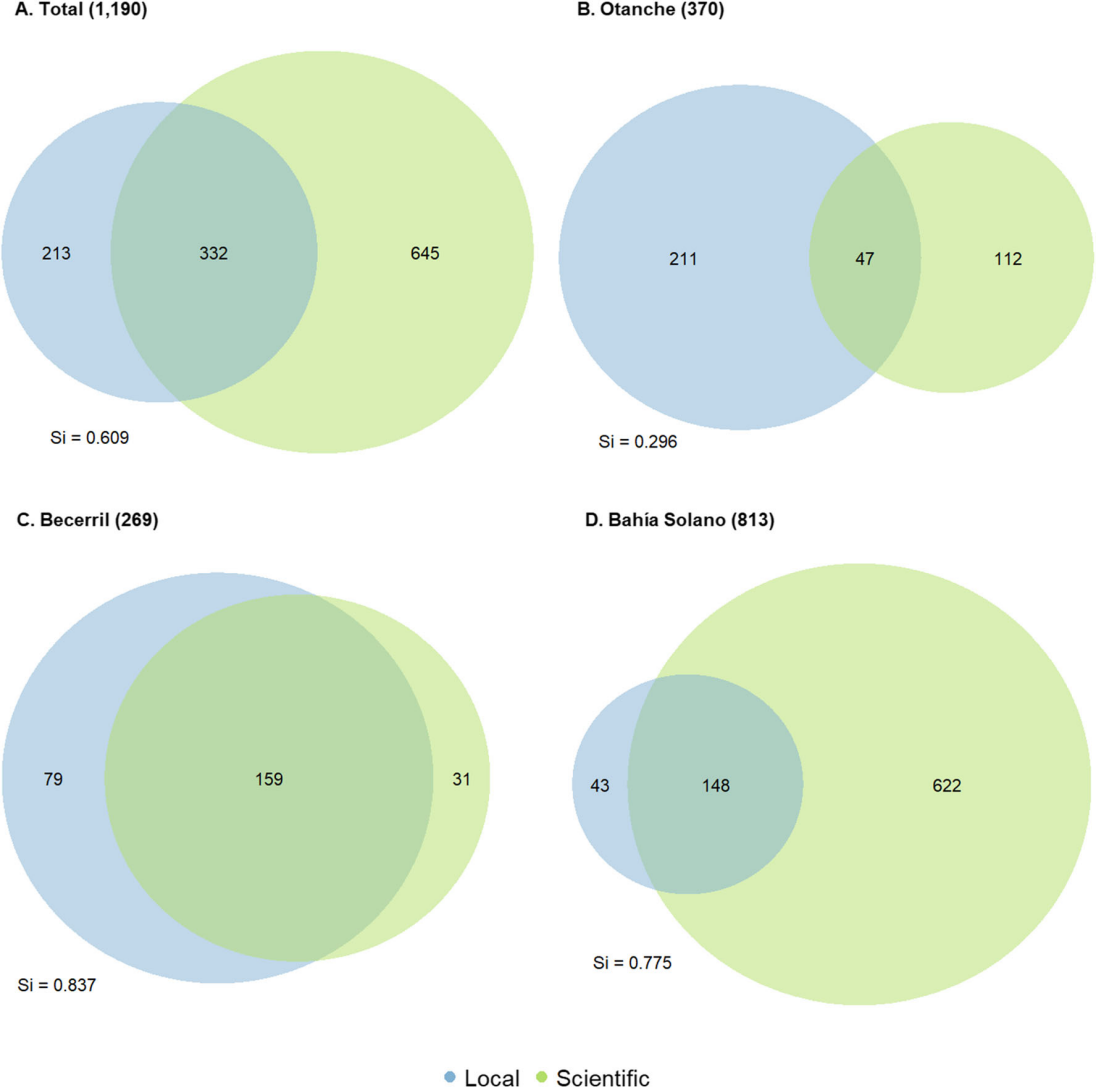
Comparison of the number of unique species reported from local and scientific knowledge sources **A** Across the three study municipalities (duplicates removed) and in **B** Otanche, **C** Becerril, **D** Bahía Solano. Figures in brackets indicate the total number of unique species in each location. *Si* Szymkiewicz–Simpson similarity index

Georeferenced data downloaded from GBIF, BIEN and JBB reported a total of 977 useful plant species based on scientific knowledge, represented by 2649 records. Combining data from local and scientific knowledge gave 1190 unique species across the three municipalities, with 27.9% overlap (*n* = 332) (Fig. 4a).

### Richness and composition

Richness and composition of useful plant species varied between the three study areas, both in absolute terms, as well as relative differences reported by scientific and local knowledge sources (Fig. 4). In Otanche and Becerril, higher useful plant richness was reported based on local knowledge sources (258 and 238 species, respectively) compared to scientific knowledge (159 and 190, respectively) (Fig. 4b, c). However, in Bahía Solano, where total species richness was the highest, the number of species reported from scientific sources (770) far outnumbered those from focus groups and walks-in-the-woods (191).

The degree of resemblance between the species lists generated by each knowledge source differed between locations. Otanche showed greatest divergence between scientific and local knowledge and was the only study area where similarity indices were lower than critical values (Si = 0.315). Becerril showed the greatest overlap between species reported from each knowledge source (Si = 0.837).

### Use types

**Fig. 5:**
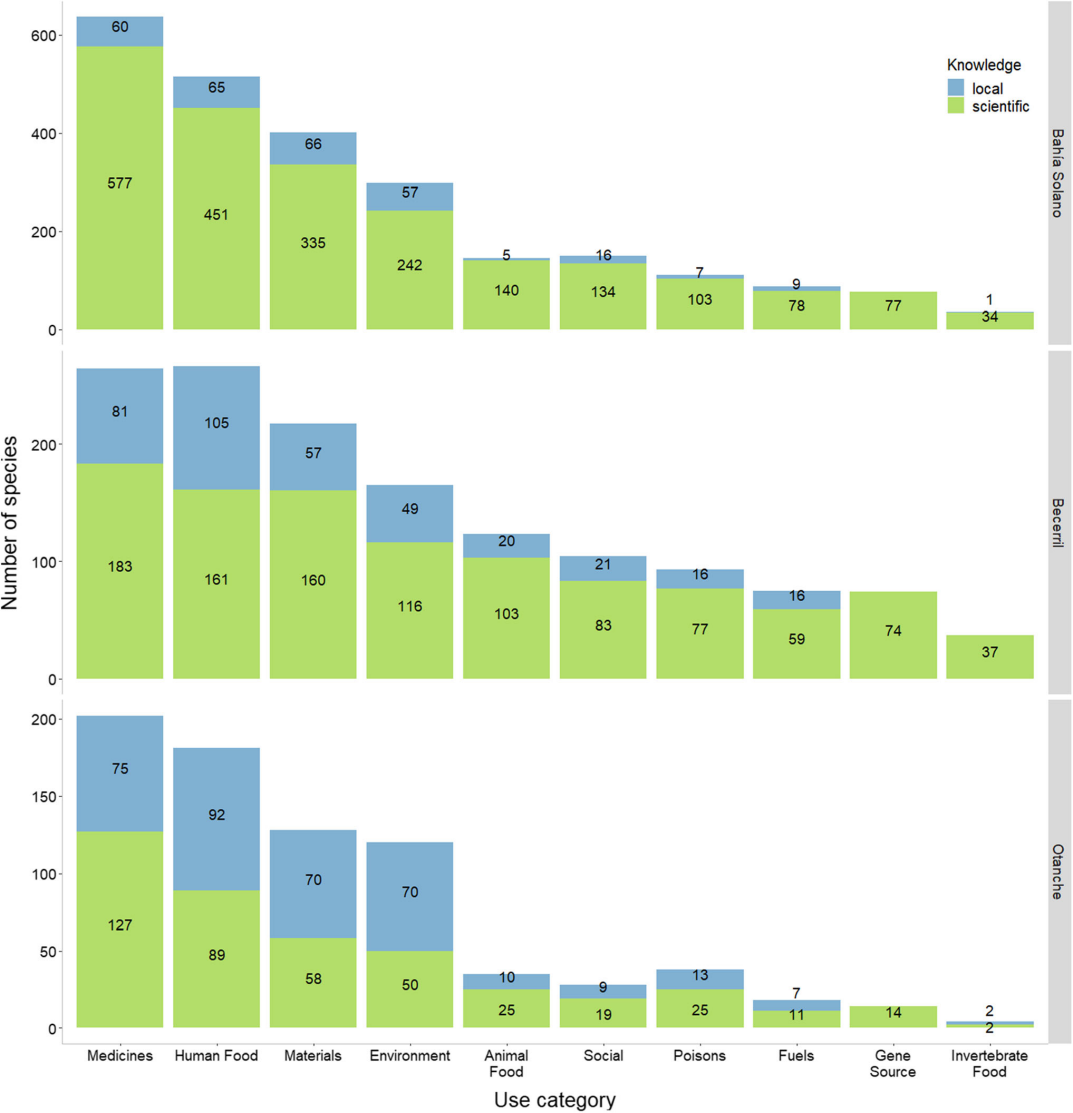
Number of species reported per use category from local and scientific knowledge sources in each municipality. Presented in descending order of total number of species reported within each category (some species were reported in multiple use categories and/or both knowledge sources)

In many cases, multiple uses were reported for a single species. Across the study areas, the total number of unique Level 1 uses was 849 from local knowledge and 2973 from scientific knowledge. This resulted in a mean of 1.6 and 3.0 uses per species from each source, respectively. From scientific knowledge, the highest proportion of reported uses across the study areas were medicinal (25.5%, *n* = 757), reflected in all three locations (Fig. 5). Meanwhile, combined local knowledge sources mainly reported plants used as human food (23.32%, *n* = 198), despite the results from Bahía Solano where most locally reported uses were materials. No plants were reported as being gene sources based on local knowledge, compared to 124 from scientific sources.

Of the 332 useful plant species reported from both knowledge sources (Fig. 4), there were 99 instances where uses reported from local knowledge were not reflected in the scientific knowledge reviewed. The majority of these fell under environmental uses (45.5%), followed by materials (14.1%), and most originated from participants in Bahía Solano and Becerril. Conversely, a total of 1054 use types reported for these species from scientific knowledge were not reflected in local knowledge in any of the three study areas. These were primarily medicinal uses (17.2%) and most commonly for species with records in Becerril.

### Important Plant Area approaches

Semi-structured interviews were conducted with 31 people in Otanche (52% men; 48% women). Most participants’ highest level of education was primary (45%) or secondary (42%), with 10% having attended university and 3% having no formal education. Nearly all participants relied on multiple occupations, with farming being the most common primary occupation (55%). While most respondents were recruited from focus groups, eight had no previous involvement with the UPFC. All respondents had relevant knowledge on the use of wild or cultivated plants in the area. However, interview results showed that respondents’ perception of the prevalence of plant use in Otanche was conversely correlated with level of education (Supplementary Figure S1). Significant correlation was not found with any other socio-demographic variable.

The presence of natural water sources was viewed as the most important factor in identifying areas for plant conservation, followed by plant richness, presence of threatened species and presence of useful plants (Table 1). Water source presence is not an IPA criterion and was added as a response option following pilot interviews and findings from focus group activities. Respondents differed in opinion on how plant conservation areas should be managed; the top two most frequently chosen options were allowing for sustainable use and strict conservation protection (Table 1). Conversely, there was strong agreement to the open question “who do you think should manage these areas?”, with 81% of responses focusing on community management. This was either viewed on a very localised level, with suggestions that management should be led by *campesinos* (local farmers) “who know the area”, or as part of a community network, with a need to “bring the *fincas* (properties) together” and create “community agreements between residents”. Some highlighted that local community management should be undertaken alongside external support, with one respondent explaining that “we have an interest in this [plant conservation], but [other] entities have much more knowledge and competence, we … need their support”.

**Table 1:** Respondent views on how plant conservation areas should be identified and managed in Otanche (based on responses to multiple choice questions by 31 participants)

		*N*	Percent	Percent of cases
How plant conservation areas should be identified	Water sources	16	37.2	51.6
	Plant richness	11	25.6	35.5
	Threatened species	9	20.9	29.0
	Useful plants	5	11.6	16.1
	Recreation value	2	4.7	6.5
	Total	43	100.0	138.7
How plant conservation areas should be managed	Sustainable use	15	38.5	51.7
	Strict protection	7	17.9	24.1
	Education	6	15.4	20.7
	Research	6	15.4	20.7
	Don’t know	5	12.8	17.2
	Total	39	100.0	134.5

## Discussion

In this study, we drew on both scientific and local knowledge to identify a total of 1190 species with reported human uses across three municipalities in Colombia. Both local and scientific knowledge sources provided unique information on useful plants which can support better-informed conservation prioritisation for a biological group whose value and threats are increasingly recognised in global conservation efforts (Khoury et al. 2019; Reyes-García and Benyei 2019). However, the extent to which each knowledge source relatively contributed to understanding species richness, composition and use types varied greatly between Bahía Solano, Becerril and Otanche. In addition, the factors perceived by participants in Otanche as most important for area-based conservation differ from global IPA criteria. In the following sections we discuss potential reasons for these differences, highlighting strengths and limitations of this study, and factors affecting the integration of multiple knowledge systems to support the conservation of useful plants in spatial conservation prioritisation.

### Importance of scale

Combined results across our study areas showed that scientific knowledge contributed a higher number of useful plant species, reported uses and mean number of uses per species than local knowledge (Figs. 4A, 5). This can be attributed to a variety of factors, primarily linked to the scales of the observations (Gagnon and Berteaux 2009).

Firstly, scientific knowledge sources drew on information across a longer temporal scale. The origin of literature contributing to the Checklist of Useful Plants of Colombia (CUPC) and occurrence records used to determine CUPC presence stretch over many decades (Diazgranados et al. 2022a). This contrasts with local knowledge sources based on fieldwork in 2021 and 2022. Temporal considerations are particularly important given the well-documented decrease in traditional ecological knowledge among indigenous and rural communities (Gómez-Baggethun and Reyes-García 2013). This was also mentioned by study participants, with the knowledge they shared unlikely to reflect plant uses over the same timescale.

Secondly, global sources formed the basis of scientific knowledge on plant uses rather than only uses reported within geographical boundaries of our case study areas (Diazgranados et al. 2022a). With use often specific to certain habitats and cultures, many uses documented in the CUPC may not have been practised in these municipalities (Cámara-Leret and Dennehy 2019). This raises a pertinent question regarding IPAs and other area-based conservation of useful species: should areas targeting socially, economically and culturally important species be identified based only on the presence of locally relevant uses, or on all useful species? The former approach decreases the extent that the burden of global conservation goals are placed on local communities and could help achieve participatory conservation-through-use and biocultural conservation (WWF-Colombia 2008). However, with underutilised plants providing potential solutions to global challenges, protecting them beyond their range of use may help to maintain genetic diversity and support future adaptation (Borrell et al. 2019). It is also important to recognise that ILK is dynamic. Alongside the loss of traditional ecological knowledge, societies continuously generate new knowledge (Gómez-Baggethun and Reyes-García 2013).

### Importance of context and study limitations

Despite the greater temporal and geographical scope of our scientific knowledge sources, local knowledge still added novel insights on useful plants in all study municipalities (Figs. 4 and 5). This supports findings that the combination of knowledge systems provides the richest understanding of biodiversity and highlights the extent of gaps in scientific datasets commonly used for conservation planning (Holland et al. 2012; Torrents-Ticó et al. 2021). However, the degree of resemblance or divergence between knowledge sources varied greatly between municipalities.

The socio-ecological context and history of research in Bahía Solano can partly explain the greater richness reported from scientific compared to local knowledge sources, with results most closely aligned to scenario F in our interpretative key (Fig. 3). As a global biodiversity hotspot, the Chocó has long attracted biologists, with high numbers of botanical studies compared to other Colombian departments (Arbeláez-Cortés 2013). Rich biodiversity combined with high scientific effort has resulted in more CUPC species with documented occurrences per unit area. In addition, traditional knowledge has decreased in the Chocó (Cámara-Leret et al. 2016), with our contemporary snapshot of local knowledge likely to reflect this.

In Otanche, local knowledge reported greater useful plant richness than scientific sources (most closely aligning with scenarios D and G in Fig. 3), while species numbers were relatively even between knowledge sources in Becerril (between scenarios B and E in Fig. 3). Boyacá and Cesar have both attracted few botanical studies (Arbeláez-Cortés 2013), resulting in gaps in scientific occurrence records for plants which are present and known to local people. The reason why the difference between knowledge sources is more pronounced in Otanche than Becerril may be partly linked to the higher biological diversity of natural ecosystems in Otanche compared to the degraded tropical dry forests of Becerril (Norden et al. 2021). Otanche’s greater species richness means that higher sampling efforts are required to scientifically document the same proportion of existing species. As noted by Gagnon and Berteaux (2009), the degree of overlap in local and scientific knowledge is likely to increase as scientific research progresses, especially as the basis of most scientific knowledge on plant uses is ILK. In addition, we had an uneven number of participants in each municipality, with greater representation in Otanche. This represents a limitation to our study, in part caused by travel constraints across Colombia at the time, as well as other factors discussed below.

Further limitations associated with our study help to explain the comparative differences. In Bahía Solano, security and travel constraints resulted in fewer focus group participants and lower geographic coverage (Fig. 2). While all participants had experience of plant use, focus groups were undertaken in Afro-Colombian coastal communities where small-scale fishing is the primary economic activity. Meanwhile, most participants in Otanche and Becerril identified as *campesinos*, defined by Granados (2020) as an intercultural subject “involved in direct work with the land and nature; immersed in forms of social organisation”. The lower direct connection with land may partly explain why useful species reported by participants in Bahía Solano reflected a lower proportion of total richness. In addition, this study was the first time researchers on the UPFC worked with communities in Bahía Solano, in contrast to previous studies involving local communities in Otanche and Becerril. Trust is vital in conservation and development involving ILK (Tuxill and Nabhan 1998). This takes time to build and may have resulted in fewer people attending workshops and participants in Bahía Solano being more guarded. The historical marginalisation of the Chocó and ongoing armed conflict potentially added to this, with a strong tradition of collective organisation and low state trust (Rodríguez Iglesias et al. 2022).

Peace and armed conflict affect biological resource use in many ways. In addition to impacts on sustainable use summarised by IPBES (2022), we found that disruption of traditional institutions impact knowledge. With a locally initiated peace process in the 1990s, Otanche represented the case study area with the most mature peacebuilding process. Communities have had longer to redevelop relationships and mechanisms for knowledge-sharing, with examples of community-led natural resource management (Mosulishvili 2016). Coupled with historically low scientific studies, this may explain the dissimilarity in species composition between local and scientific sources. In contrast, Becerril showed the highest overlap. The area has been more recently affected by armed conflict (Eufemia et al. 2021) and numerous participants who had only recently returned following displacement reported that the workshops of the UPFC and other local sustainable development organisations were some of the first opportunities for neighbouring *campesinos* to come together. Resultantly, current knowledge may have reflected the scientific understanding shared during workshops rather than traditional knowledge of the area. This highlights the importance of building capabilities for local communities to develop their own knowledge base and allowing for continued exchange between knowledge systems (Charles 2021).

Despite the drivers of “under-sampling local knowledge” and “lack of trust” being relevant in Bahía Solano (Fig. 3), it was the municipality where the most previously undocumented uses for species in the CUPC were reported. This may be due to the engagement of communities not traditionally involved in ethnobotanical studies. In addition, findings should be placed in their historical context. Botany as a Western scientific discipline began with a medical focus in the sixteenth and seventeenth centuries, then developing into the broader concept of ‘economic botany’, with the term ‘ethnobotany’ only emerging at the turn of the twentieth century (Cornish and Nesbitt 2014). This demonstrates the discipline’s long focus on plants with potential economic importance and explains the dominance of medicinal uses in scientific knowledge sources. Previously undocumented plant uses arising from local knowledge were primarily environmental, as ornamentals or for shade and livestock shelter (Cook 1995), uses with local significance but limited economic interest.

Our application of the WCUP use categories contrasts with the generally recommended use of emic categories in ethnobotany (Martin 1995). While our etic approach allowed for standardisation and data collection relevant to spatial conservation planning, some categories—such as gene sources—can only arise from scientific knowledge. In the division of plant uses applied by Tuxill and Nabhan (1998) into ethnobotanical resources (directly used for consumption or income generation) and phytogenetic resources (indirectly used as sources for a variety of industries), newly documented knowledge on the former is likely to be generated from local sources, while scientific sources are more likely to directly contribute to the latter. It is therefore important to recognise both the limitations and unique contributions of each system.

### Area-based plant conservation approaches

Our focus on plant species richness and use was based on IPA guidelines (Darbyshire et al. 2017; Diazgranados and Castellanos-Castro 2018). However, interview results in Otanche showed that participants valued the presence of water sources more highly than the most commonly applied IPA criteria of threatened species and exceptional richness, or the presence of useful plants. This response option was added to our interview following a pilot study and focus groups, where water clearly featured in participants’ perception of conservation, reflecting findings by Feeney (2021) on understanding of biodiversity in Colombia.

Beyond the repeated core sentiment that “*agua es vida*” (“water is life”), several respondents tied the health of natural water sources with that of plants, with one explaining that “many plants protect water, and they need water too”. This perception may be heightened by locally relevant environmental conflicts. Ulloa et al. (2022) place water at the centre of demands for environmental justice in Colombia, with large-scale mining—a key economic activity in Otanche—shown to result in fragmented or absent water governance. A respondent who listed water sources, useful plants and recreational value as the three most important factors for identifying plant conservation areas highlighted the significance of making nature relevant to achieve conservation success: “the others [threatened species and richness] are important too, but if you don’t have this sentiment [of relevance], it is difficult to win”. This was reflected in local perceptions of how and by whom plant conservation areas should be managed. Participants’ strong preference for allowing sustainable use mirrors the “conservation-through-use” concept, while the overriding response that conservation areas should be locally managed matches findings that sustainable outcomes are more likely through actively working with local communities (Kor et al. 2021). This was coupled with numerous participants’ calls for training and support from authorities and “experts”, supporting findings that pathways for sharing education and knowledge are crucial in achieving successful community conservation (Charles 2021).

In not automatically conferring legal protection, IPAs are distinct from general definitions of protected areas, with guidelines highlighting their potential to drive community-led management. Despite this, our findings show a mismatch between local priorities in Colombia and the global and Colombian IPA criteria, designed by academics and conservation practitioners (Darbyshire et al. 2017; Diazgranados and Castellanos-Castro 2018). This points towards the need to move participatory approaches beyond community-based conservation and data collection, to include plural worldviews in designing global conservation programmes themselves, especially in the light of criticisms of large-scale priority mapping (Wyborn and Evans 2021). IPA criteria closely reflect those of other global conservation programmes such as Key Biodiversity Areas (KBA), making the need to bridge local and scientific knowledge of growing importance if we are to meet global conservation goals to conserve 30% of earth for biodiversity “for the benefit of people and nature” by 2030 (CBD 2022; Reyes-García et al. 2022).

### Supplementary Information

Below is the link to the electronic supplementary material.

Supplementary file1 (PDF 252 kb)

## Data Availability

The data supporting this article are openly available from Figshare at https://doi.org/10.6084/m9.figshare.23519892.
