# Supplementary material for: Bridging local and scientific knowledge for area-based conservation of useful plants in Colombia
Source: Ambio. 2023 Oct 12;53(2):309–23. doi: 10.1007/s13280-023-01921-5 (PMC10774498; doi:10.1007/s13280-023-01921-5)

Ambio

Supplementary Information

This supplementary material has not been peer reviewed.

Title: **Bridging local and scientific knowledge for area-based conservation of useful plant in Colombia**

Authors: Laura Kor, Mateo Fernández-Lucero, Diego Arturo Granados Flórez, Terence P Dawson, Mauricio Diazgranados

This document includes:

Table S1. Definitions of Level 1 plant use categories and summary of their relevant Level 2 states and Level 3 descriptors based on Diazgranados et al. (2020) and Cook (1995) [page 2]

Interview Information S1. Content of the information sheet provided to participants ahead of semi-structured interviews. A version of this was also provided for focus group and ethnobotanical survey participants [pages 3-5]

Interview Information S2. Content of consent form completed by participants prior to undertaking semi-structured interviews [page 6]

Interview Information S3. Relevant sections of semi-structured interview undertaken in Otanche, Boyacá in July 2022. Interviews were conducted in Spanish, with English translation provided here [pages 7-8]

Supplementary Figure S1. Relationship between level of formal education and perception of plant use prevalence in Otanche. Based on interview question "How common is it for people in Otanche to use wild plants? Where 1 is extremely rare and 5 is extremely common" [page 9]

Table S1. Definitions of Level 1 plant use categories and summary of their relevant Level 2 states and Level 3 descriptors based on Diazgranados et al. (2020) and Cook (1995)

**Level 1 category ***	**Description**	**Relevant Level 2 states and Level 3 descriptors #**
Animal food	Forage and fodder for vertebrate animals	*Animal food types include:* hay, silage, concentrates, etc. for forage, grazing, fodder
Environmental uses	Examples include intercrops and nurse crops, ornamentals, barrier hedges, shade plants, windbreaks, soil improvers, etc.	*Specific environmental uses include:* agroforestry, boundaries, barriers, support, soil improvers (e.g. nitrogen fixers, soil moisture conservers), pollution controllers, ornamentals, firebreaks, shade/shelter
Fuels	Wood, charcoal, petroleum substitutes, etc. – separated from materials because of their importance.	*Fuel uses include:* cooking, heating, lighting, vehicle fuels
Gene sources	Wild relatives of major crops which may be valuable for breeding programs.	*Beneficial genetic traits include*: disease resistance, pest resistance, high yields, cold tolerance, waterlogging tolerance
Human food	Food and beverages for humans only, including food additives.	*Situations when food is used include:* famine, staple, non-staple, ceremonial, snack, weaning, regular, and diabetic foods<br>*Preparations include:* raw, savoury, cereal and starch-based (e.g. cakes, breads, etc.) dairy and dairy-like (cheese, etc.), condiments, pickles, pastes, beverages (alcoholic and non-alcoholic)<br>*Food additive types include:* colourings, fermenting agents, flavourings, preservatives, sweeteners
Invertebrate food	Plants eaten by invertebrates which are useful to humans (e.g. silkworms)	*Useful invertebrate types include:* silkworms, lac insects, edible insects, dye-containing insects,
Materials	Woods, fibers, cork, cane, tannins, latex, gums, etc. and their derived products.	*Material types include:* fibres, cane, wood, gums, tannins, lipids, waxes<br>*Products used in include:* buildings, cleansers, clothing, coatings (e.g. paints, waterproofers), constructions, containers, cosmetics, fasteners, fishing equipment, floors, furnishings, illuminants, machines, musical instruments, printed material, perfumes, personal items roofs, ropes, sports equipment, tools, toys, vehicles, walls
Medicines	Both human and veterinary.	*Medicinal uses include for:* Unspecified Medicinal Disorders, Abnormalities, Circulatory System Disorders, Digestive System Disorders, Endocrine System Disorders, Ill-Defined Symptoms, Immune System Disorders, Infections/ Infestations, Inflammation, Injuries, Mental Disorders, Metabolic System Disorders, Muscular-Skeletal System Disorders, Neoplasms Nervous System Disorders, Nutritional Disorders Pain, Poisonings, Pregnancy/ Birth/ Puerperium Disorders, Respiratory System Disorders, Sensory System Disorders, Skin/ Subcutaneous Cellular Tissue Disorders
Poisons	Plants which are poisonous to vertebrates and invertebrates, both accidentally and usefully (e.g., for hunting and fishing).	*Poison uses include*: hunting, fishing, plant pest control, livestock pest control, house pest control, timber protection
Social uses	Plants used for social purposes not definable as food or medicines. Such as smoking materials, hallucinogens and psychoactive drugs, contraceptives and abortifactants, and plants with ritual or religious significance.	*Social use types include:* smoking materials and drugs, antifertility agents, 'religious' uses (e.g. sacred plant)

** Definitions follow the World Checklist of Useful Plants, which combined the 13 Level 1 uses of Cook et al. into 10 categories (Diazgranados et al., 2020)*

[#] In Cook et al (1995), how Level 2 states are derived depends on the Level 1 use. E.g, for food, this refers to the part of the plant, while for materials this is the type of material obtained. We have provided a summary which mixes elements of level 2 states and level 3 descriptors which were most relevant when categorising uses in this study

Interview Information S1. Content of the information sheet provided to participants ahead of semi-structured interviews. A version of this was also provided for focus group and ethnobotanical survey participants.

Consentimiento informado sobre la participación en la investigación 'Plantas y Hongos Útiles de Colombia'

Ethical Clearance Reference Number: LRS/DP-21/22-22709 | Versión 3 | 20/06/22

Me gustaría invitarle a participar en este proyecto de investigación que forma parte de mi investigación de doctorado. Antes de decidir si desea participar, es importante que comprenda por qué se realiza la investigación y en qué consiste. Por favor lea atentamente la siguiente información y si lo desea, discútala con otras personas. Pregúnteme si hay algo que no le queda claro o si desea más información.

¿En qué consiste esta investigación?

Mi investigación es parte del proyecto Plantas y Hongos Útiles de Colombia. Los objetivos del proyecto son incrementar, consolidar y hacer accesible el conocimiento sobre las plantas y hongos útiles para el beneficio de todos, promover un mercado para especies nativas y sus productos de alto valor, y motivar el uso sostenible de la biodiversidad mientras se protegen los recursos naturales. El enfoque de mi doctorado está en la conservación de las plantas útiles.

¿Debe usted participar en esta investigación?

Su participación es voluntaria y si es su decisión personal, usted puede abandonar la investigación en cualquier momento sin justificarse. Debido a que no se está recolectando información personal, usted no podrá retirar su consentimiento después de completar las encuestas y/o entrevistas.

¿Qué le solicitamos durante la investigación?

Este estudio no ofrece ningún tipo de compensación económica por participar, es totalmente voluntario. Lo(a) invitamos a participar en algunas de las actividades que enlistamos a continuación:

- Entrevistas relacionadas con el conocimiento, uso y conservación de plantas y hongos (20-40 min)
- Participar en talleres interactivos en los que se discutirán el conocimiento, uso y conservación de plantas y hongos útiles nativos, procesos de gobernanza y otros temas relacionados (medio día)
- Registrar su participación a través medios audio y fotográficos

¿Cómo trataremos su información?

La información recogida será empleada para publicaciones científicas, reportes y plataformas de conocimiento en línea de dominio público. Los datos registrados a través de encuestas, talleres y conversaciones serán anónimos. Por favor mantenga toda la información de los talleres de manera confidencial, y sea consciente de que no podemos garantizar la confidencialidad de la información que se comparte en los talleres con otras personas.

Cualquier dato personal recopilado independientemente de la encuesta anónima como se describe en otra parte de esta hoja de información se procesará según los términos de la ley de protección de datos del Reino Unido (incluido el Reglamento General de Protección de Datos del Reino Unido (RGPD del Reino Unido) y la Ley de Protección de Datos de 2018). Los datos se almacenarán de forma segura en SharePoint hasta la finalización de mi proyecto de doctorado (octubre 2023). Si desea obtener más información sobre cómo se procesarán sus datos según los términos de las leyes de protección de datos del Reino Unido, visite: https://www.kcl.ac.uk/research/support/research-ethics/kings-college-london- declaración-sobre-el-uso-de-datos-personales-en-investigación

¿Cómo se financia el proyecto?

Soy estudiante en King's College London y mi doctorado es financiado por el 'Natural Environment Research Council' (NERC) del Reino Unido. El UPFC es apoyado por un subsidio de Desarrollo Profesional y

Compromiso a través del Fondo Newton-Caldas. El Real Jardín Botánico de Kew y el Instituto Humboldt estarán ejecutando el proyecto en las áreas priorizadas de Becerril, Otanche y Bahía Solano.

¿Qué más debo saber?

Usted puede realizar preguntas sobre la investigación, la privacidad y el manejo de sus datos personales a Laura Kor, L.Kor@kcl.ac.uk, Mauricio Diazgranados, M.Diazgranados@kew.org. Puede retirar sus datos del proyecto hasta el 4 de Agosto poniéndose en contacto con Laura Kor. Después de esta fecha no será posible retirar datos porque serán procesados y anonimizados.

¿Inquietudes relacionadas con sus derechos como participante en la investigación? Puede contactar al Comité de Ética del King's College London, rec@kcl.ac.uk

---

Declaro que he leído la información sobre la investigación. Manifiesto que entiendo de qué se trata, qué se solicita de mi parte, cómo serán manejados los datos que aquí voy a suministrar y cuáles son mis derechos como participante. Expreso que mi participación es voluntaria. Por favor reitere que da su consentimiento para participar en esta investigación.

___ Si, doy mi consentimiento para participar

___ No, no doy mi consentimiento para participar

Firma: ___________________ Nombre: ____________________

Fecha: _____________________

Interview Information S2. Content of consent form completed by participants prior to undertaking semi-structured interviews.

Formulario de consentimiento para participantes en proyectos de investigaciones

Complete este formulario cuando ha leído la hoja de información y/o ha escuchado una explicación sobre la investigación

**Título: Plantas y Hongos Útiles de Colombia**	
**Número de referencia de revisión ética: LRS/DP-21/22-22709**	**Version 2 | 20/06/22**
	Marca o inicial
1. Confirmo que he leído y entendido la hoja de información del 20/06/22 para el proyecto anterior. He tenido la oportunidad de considerar la información y hacer preguntas que han sido respondidas a mi satisfacción.	
2. Doy mi consentimiento voluntario para participar en este proyecto y entiendo que me puedo rehusar a participar y retirar del proyecto, sin tener que dar una razón hasta el 04/08/22.	
3. Doy mi consentimiento para el procesamiento de mi información personal como se me ha explicado en la hoja de información. Entiendo que tal información será manejada bajo los términos de la ley de protección de datos del Reino Unido (incluido el UK General Data Protection Regulation (UK GDPR) y el Data Protection Act 2018).	
4. Entiendo que mi información puede estar sujeta a revisión por parte de personas del Colegio con fines de auditoría.	
5. Acepto mantener la confidencialidad de las discusiones de los talleres y entiendo que no se puede garantizar la confidencialidad de información que comparto en los grupos.	
6. Acepto que se pueden compartir mis datos con transcriptores de terceros que hayan firmado un acuerdo de confidencialidad	
7. Acepto que el equipo de investigación puede usar mis datos para otras investigaciones en el futuro.	
8. Doy mi permiso para grabar en audio mi participación en la investigación.	
9. Doy mi permiso para fotografiarme durante de mi participación en la investigación.	
10. Entiendo que la información que he enviado se publicará en reportes, trabajos académicos, presentaciones y artículos en línea.	
11. Entiendo que se mantendrá la confidencialidad y anonimato, y no será posible identificarme en los resultados de la investigación.	

________________ ______________ _______________ _______________

Nombre del participante **Fecha** **Firma** **Número de teléfono**

________________ ________________ _______________

Nombre del investigador **Fecha** **Firma**

Interview Information S3. Relevant sections of semi-structured interview undertaken in Otanche, Boyacá in July 2022. Interviews were conducted in Spanish, with English translation provided here.

Participation in interview

Ensure that the Participant Information Sheet has been given or read to the participant, any questions have been answered, and written or recorded consent has been gained.

Date: ____

Village/community: __________________________________

Interviewer name: ___________________________________

Survey number: _____________________________________

Participant information

1. Socio-demographic information:

    a. Gender:
        M / F / Other
    b. Age group
        <25 / 25 – 40 / 41 – 60 / >60
    c. Highest level of Education

    d. Primary, secondary and tertiary occupations

2. Have you previously participated in any UPFC workshops or expeditions?

    Yes / No / Don't know

Note: as many as possible of these questions should be covered, however not all may be relevant to the respondent, so allow for flexibility. If the informant raises an interesting point, the interviewer should attempt to explore this further. Points in brackets are prompts or follow up questions if needed

Topic 1: Plant use and conservation

3. On a scale of 1 to 5, how common is it for people in Otanche to use wild plants? Where 1 is extremely rare and 5 is extremely common. (i.e., to collect plants for medicine, food, building materials, timber, etc.?)

    1 ---- 2 ---- 3 ----- 4 ----- 5

4. If you collect wild plants, have there been changes in their abundance in recent years?

    Yes / No / Don't know

    a. If so, what has changed? (e.g., changes in: abundance of plants; types of plants; timing of harvest; use / demand; etc.)

    b. What has caused these changes? (e.g., land-use change, overharvesting, climate change, pollution, loss of traditional knowledge/practices etc.)

5. What do you think is the biggest risk to useful plants in the area?

6. If you were in charge of protecting plants, what would be your priority for conservation? (e.g., public education and awareness, protected areas, commercialise species to increase value of conservation, set quotas or limits, ban harvesting certain plants / in certain seasons, promote cultivation, more research, etc.)

7. The Important Plant Areas (IPA) programme aims to protect the most important sites for plant conservation in the world. We are trying to identify IPAs in Colombia and would like your opinion on this.

    a. How do you think plant conservation areas should be chosen?

- ☐ presence of threated species
- ☐ species richness (many different species)
- ☐ importance for useful plants
- ☐ importance for water sources
- ☐ importance for recreation
- ☐ other _______________________

b. How do you think plant conservation areas should be managed?

- ☐ strict protection with no entry
- ☐ entry allowed for research
- ☐ entry allowed for education
- ☐ entry allowed for recreation
- ☐ sustainable plant harvesting allowed
- ☐ other ________________________

c. Who do you think should manage these areas? (*Prompts if required*: local communities; municipality authorities; national government; NGOs; researchers; other)

Topic 2: Changes in useful plants

[This section included questions on changes in the use and abundance of specific species in the municipality]

Any other comments

Supplementary Figure S1. Relationship between level of formal education and perception of plant use prevalence in Otanche. Based on interview question "How common is it for people in Otanche to use wild plants? Where 1 is extremely rare and 5 is extremely common"

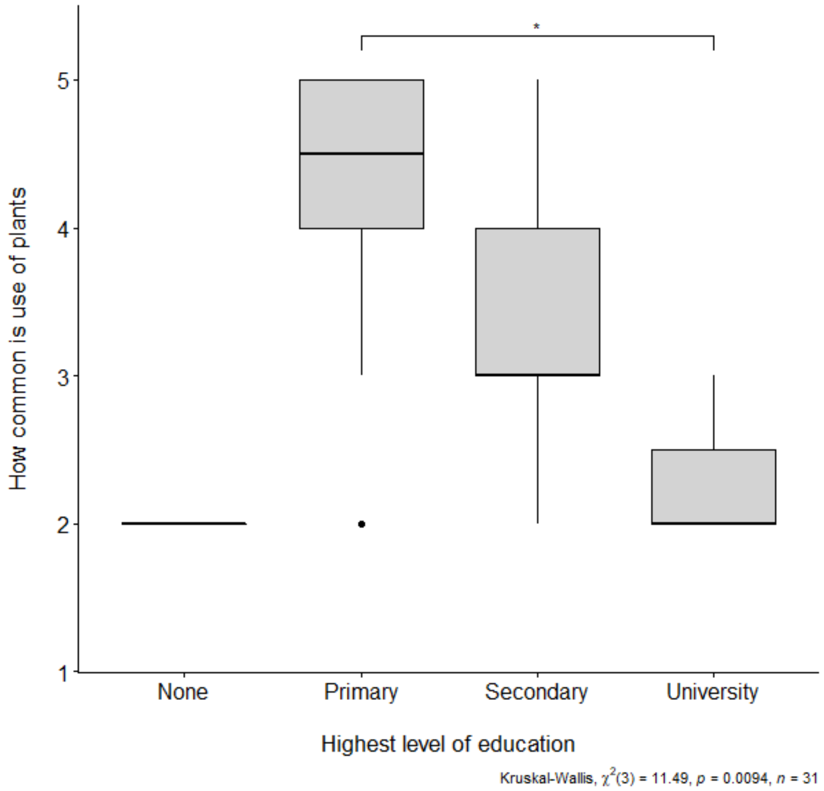

Supplement: Supplementary file 1 — Supplementary file1 (PDF 252 kb) [file 13280_2023_1921_MOESM1_ESM.pdf]
